# Multi-criteria solar power plant siting problem solution using a GIS-Taguchi loss function based interval type-2 fuzzy approach: The case of Kars Province/Turkey

**DOI:** 10.1016/j.heliyon.2024.e30993

**Published:** 2024-05-10

**Authors:** Gokhan Sahin, Ibrahım Akkus, Ahmet Koc, Wilfried van Sark

**Affiliations:** a Copernicus Institute of Sustainable Development, Utrecht University, Princetonlaan 8A, 3584 CB, Utrecht, the Netherlands; b Igdir University, Engineering Faculty, Electrical and Electronical Engineering Department, Igdir, Turkey; c Dicle University, Vocational School of Technical Sciences, Park and Garden Plants Department, Diyarbakır, Turkey

**Keywords:** Environmental factors, Suitability solar energy map, Geographical information systems (GIS), Multi-criteria decision making, Fuzzy logic, Interval Type-2 fuzzy sets, Taguchi loss function

## Abstract

The determination of the areas where the solar power plant will be installed is of great importance for the performance of the solar power plant. Solar and hydroelectric energy are the most widely used renewable energy sources in Kars province. Site selection for these power plants is an important factor in terms of reducing the installation cost of the solar power plant and achieving maximum efficiency during operation. Determining the areas where the power plants will be installed is a very complex and difficult to analyse spatial decision making problem. In this study, firstly GIS is used as a mapping method to obtain the locations of both solar power plants in Susuz, Arpaçay, Akkaya, Kars city centre, Selim, Digor, Kağızman and Sarıkamıș districts of Kars province and then Taguchi loss function based interval type-2 fuzzy approach is applied to the problem. In order to obtain more accurate results, the results of the two methods (GIS and Taguchi loss function based interval type-2 fuzzy approach) were also compared. According to the solar power plant map obtained, it was determined that the total area of suitable areas is 78600 km^2^.

## 1 Introduction

The global population has a persistent upward trend. The rise in population leads to a corresponding increase in the energy supply [1]. Researchers highlight that the finite and scarce reserve of fossil resources is expected to diminish within a span of 30–40 years [2]. The depletion and limited availability of fossil resources has prompted individuals to seek alternative energy sources. Indeed, the need for electricity-based energy has steadily risen. Technological progress over the past century has led to a significant growth in the utilization of electricity. Globally, the consumption of electricity has doubled approximately every 14.5 years [3,4]. In reality, the infrastructure needed for electricity generation is primarily supplied by fossil fuels. When examining global energy production data, it is evident that the majority, accounting for 60 %, is derived from fossil fuels. The extensive utilization of fossil fuels exacerbates the strain on the climate and environment [5,6]. In response to the escalating environmental deterioration and associated challenges, a directive has been issued to promote the utilization of renewable energy sources as a means to mitigate climate change [7]. Simultaneously, the countries who embraced this instruction influenced their policies. To ensure a sustainable environment, economic growth policies are being developed from a perspective of sustainable development. These policies consider the environment and natural life, taking into consideration the irreversible decline in natural life and environmental degradation [8]. Indeed, in order to achieve the objectives of these regulations, it is imperative to conduct an analysis of the locations where renewable energy systems will be implemented. The advancement of technology is currently enabling these analyses at a faster pace. Software packages designed for Geographic Information Systems (GIS) and remote sensing infrastructures offer significant assistance, particularly in the development of renewable energy power plants. Upon reviewing the literature, researchers have presented the incorporation of renewable energy on a global scale into the Geographic Information System (GIS) framework [[9], [10], [11], [12], [13]]. Simultaneously, the researchers utilized GIS and remote sensing infrastructure systems to determine land usage for solar power plants [14,15], wind power plants [[16], [17], [18]], and hybrid power plant locations [19,20], based on ecological factors. Due to the significant surge in electricity demand in Turkey, it will eventually necessitate a shift towards alternative energy sources that possess abundant potential. The demand for solar energy systems, as a form of alternative energy, is steadily rising. These systems play a significant part in addressing energy challenges [21]. Solar energy is characterized by a low carbon footprint and negligible environmental threat. Solar energy provides a highly efficient method for converting solar radiation into power. Utilizing solar technologies to harness the energy of sunshine can offer extensive energy storage alternatives in rural regions. The user's text is " [22]". Karipoglu F. et al. Charging stations fueled by sustainable energy sources are crucial for mitigating the ecological consequences of vehicles. The objective of this work is to using GIS methodology [23] to identify the optimal sites and tariffs for hydrogen filling stations, which are powered by combined solar power plants. Genc M. S et al. assessed the overall practicality of a proposed offshore wind power plant by employing Geographic Information Systems (GIS) and Multi-Criteria Decision Making (MCDM) guidance [24]. In a separate investigation, Karipoglu F. et al. employed Geographical Information System (GIS) and Multi-Criteria Decision Making (MCDM) techniques to ascertain several elements, such as wind potential, road networks, and water resources, in the development of wind power plants. The researchers then analyzed these components in relation to buffer zones [25].

This study aims to determine the solar energy potential of Kars province based on specific factors and provide the first example of a renewable energy project planned for the province. This study assessed different potential sites for the solar power plant by employing Geographic Information System (GIS) as a method for Multi-Criteria Decision Making (MCDM) to evaluate their performance. The problem has been thoroughly analyzed utilizing a Taguchi loss function based interval type-2 fuzzy methodology, combined with GIS mapping method, to identify the optimal places for solar power.

## 2 Background

This section presents the basic steps and an overview of the scientific literature on Geographic Information Systems (GIS) and Taguchi loss function based interval type-2 fuzzy approach used in the application.

### 2.1 Mapping technique, GIS

Geographic Information Systems have been variously characterized by different scientists. GIS is a comprehensive system that encompasses the acquisition, storage, control, processing, analysis, and display of spatial data related to the Earth [26]. Defined GIS as a comprehensive information system that stores, analyzes, and visually presents both spatial and non-spatial data [27]. Characterized a database as a specialized information system capable of housing attributes, activities, or events that are distributed in space and can be represented as points, lines, and areas [28]. defines GIS as a robust suite of tools used for gathering, storing, retrieving, and visualizing spatial data in the physical world. Similarly [29], characterizes GIS as a tool that acquires, saves, and manages geographic data on a global scale. It is a comprehensive system that combines, utilizes, analyzes, and displays information for a specific purpose. The user's text is " [30]." Researchers in Turkey have conducted multiple research on Geographic Information Systems (GIS) and have employed diverse definitions of GIS. As stated by Ref. [31], GIS is a comprehensive information system that collects, stores, processes, and presents both graphical and non-graphical data gathered from location-based observations in a unified manner to the user. As stated in Ref. [32]. Geographic Information System, is a computer-based system that allows for the input, analysis, and display of various types of data related to objects and events on Earth. This data is entered into the computer using real coordinates and can be presented in the form of maps, tables, and graphics. As stated by Ref. [33], GIS is a computerized system that utilizes geographic data to carry out a range of management and analysis functions. Another definition of GIS states that it enables the input of data with geographical attributes (such as climate, vegetation, landforms, population, settlement, etc.) into a computer system through digitization. This inputted data can then be used to generate new data and utilized accordingly. Interrogating, organizing, scrutinizing, and illustrating the interconnections between various elements and the outcomes achieved through the use of graphics, maps, 3D pictures, and other visual representations. The system is a computerized mapping system that relies on visualization techniques [19,34]. After the creation of the base maps, the outcome maps were generated by assigning class values individually for each usage map in the recclassify module of the Arc-GIS 10.8 package program. This process was used to build wind maps, solar maps, and combined wind + solar maps [[13], [35], [36]]. The data flow and analysis in this model are depicted in Fig. 14. The base maps were generated by integrating 12 digital elevation models (DEMs) with a resolution of 12.5 m from the Alos Polsar satellite [37]. This was done using the Spatial Analysis module of the Arc-GIS 10.8 package program. The resulting maps included slope, aspect, solar radiation, and elevation. Wind speed and air temperature maps were acquired in DEM format and generated using the Spatial Analysis module of the ArcGIS 10.8 software suite [38]. Regrettably, the available satellite data were inadequate for the research area when generating air pressure and air humidity maps. To this end, data on the yearly average humidity and pressure from 28 distinct meteorological stations located inside the Netherlands were acquired and analyzed on an individual basis. The Geostatistical Analyst module of the Arc-GIS 10.8 package application was used to transform point data into spatial data. Surface temperature maps were generated by utilizing band 10, band 4, and band 5 data from the Landsat 8 satellite and processed using the Arc-GIS 10.8 software package. The CORINE level 3 maps were simplified to level 2 and converted into a digital format using the Arc-GIS 10.8 software in land use mapping. The power lines and road lines maps were converted from raster format to a digital format using the ArcGIS 10.8 program. The distance coefficients were then calculated using the Buffer module.

### 2.2 The motivation for using a multi-criteria decision making approach

Multi-criteria decision making (MCDM) is a method that involves making judgments when there are conflicting criteria and many objectives, such as maximizing efficiency or minimizing cost. The goal is to identify the best option from a set of possibilities. MCDM approaches have been suggested for various domains, ranging from site selection to material evaluation. Various Multiple Criteria Decision Making (MCDM) methods have been documented in the literature, such as Analytic Hierarchy Process (AHP), Analytic Network Process (ANP), Technique for Order Preference by Similarity to Ideal Solution (TOPSIS), fuzzy sets and systems, and Multi-attribute Utility Technique (MAUT). The Analytic Hierarchy Process (AHP) is a widely recognized Multiple Criteria Decision doing (MCDM) method that involves doing pairwise evaluations of alternatives within a hierarchical structure [[39], [40], [41]]. Nevertheless, this approach is inadequate when uncertainty occurs as a result of the subjective evaluations made by decision makers. In addition, it is incapable of solving difficult problems when there are more than fifteen possibilities [42,43]. In this study, a total of 34 alternative helicopter engines are assessed utilizing interval type-2 fuzzy sets, which are capable of handling these challenges. Subsequently, small groups of alternatives with identical rankings are examined based on their rankings. The Taguchi loss function based upon type-2 fuzzy technique was employed to offer an accurate rating for each choice. Extensive research has been carried out in the field of selection problems, resulting in several studies and the development of various solution strategies. However, as far as the authors are aware, this study represents one of the initial instances where interval type-2 fuzzy sets are utilized for wind power plant site selection. Furthermore, Table 1 illustrates the utilization of several multi-criteria techniques in addressing the issue of siting solar power plants.

**Table 1:** Summary of literature on energy alternative selection problems.

	Study	Method	Result
[44]	Mirzaei N. (2022)	Pythagorean Fuzzy	Mirzaei, N. A Multicriteria Decision Framework for Solar Power Plant Location Selection Problem with Pythagorean Fuzzy Data: A Case Study on Green Energy in Turkey.
[45]	Kahraman C. (2024)	fuzzy sets and their AHP extension	Proportional picture fuzzy sets and their AHP extension: Application to waste disposal site selection.
[46]	Haktanır E., Kahraman C. (2024)	AHP & TOPSIS	Integrated AHP & TOPSIS methodology using intuitionistic Z-numbers: An application on hydrogen storage technology selection.
[47]	S Turk (2022)	Taguchi loss function in intuitionistic fuzzy sets	Taguchi loss function in intuitionistic fuzzy sets along with personal perceptions for the sustainable supplier selection problem.
[48]	Gottwald D., Chocholá J., Çodur M. K., Dobrodolac M.C.and Kubra Yazir K.(20224)	Z-numbers fuzzy AROMAN	Z-Numbers-Based MCDM Approach for Personnel Selection at Institutions of Higher Education for Transportation.
[49]	Taguchi G. (1987)	Taguchi	System of Experimental Design: Engineering Methods to Op-timize Quality and Minimize Costs. No. 1. c. in System of ExperimentalDesign: Engineering Methods to Optimize Quality and Minimize Costs; UNIPUB/Kraus International Publications. ISBN 9780941243001.
[20]	Şahin G., Koç A, Sark van W, (2024)	GIS-intuitionistic fuzzy	Multi-criteria decision making for solar power - Wind power plant site selection using a GIS-intuitionistic fuzzy-based approach with an application in the Netherlands.
[50]	Wiguna, K.A., Sarno, R. and Ariyani, N.F., (2016)	Multi-Criteria Decision Making Fuzzy AHP and PROMETHEE	Optimization Solar Farm site selection using Multi-Criteria Decision Making Fuzzy AHP and PROMETHEE: case study in Bali, 2016 International Conference on Information & Communication Technology and Systems (ICTS).
[51]	Suh J & Brownson J R S., (2016)	geographic information system fuzzy sets and analytic hierarchy processes	Solar farm suitability using geographic information system fuzzy sets and analytic hierarchy processes:Case study of Ulleung Island.
[52]	Solangi, Y.A., Shah, S.A.A., Zameer, H. et al. (2019)	AHP-fuzzy VIKOR	Assessing the solar PV power project site selection in Pakistan: based on AHP-fuzzy VIKOR approach.
[53]	Sánchez-Lozano J M, Teruel-Solano J, Soto-Elvira P & García-Cascales M S (2013).	Geographical information systems (GIS) and multi-criteria decision making (MCDM)	Geographical information systems (GIS) and multi-criteria decision making (MCDM) methods for the evaluation of solar farms locations: Case study in south-eastern Spain.
[54]	Geng, S., Lin, L., Zhang, L., Liu, X., Huang, Z., (2020)	intuitionistic fuzzy	Site selection framework of fishing photovoltaic hybrid project under interval-valued intuitionistic fuzzy environment.
[55]	Garni A., Hassan Z. & Awasthi, Anjali, (2017)	GIS-AHP	Solar PV power plant site selection using a GIS-AHP based approach with application in Saudi Arabia.
[56]	Garg, H., (2017)	intuitionistic fuzzy	Novel intuitionistic fuzzy decision making method based on an improved operation laws and its application.
[57]	De Santoli, L., Mancini, F., Garcia, D. A., (2019)	GIS	A GIS-based model to assess electric energy consumptions and useable renewable energy potential in Lazio region at municipality scale.
[58]	Colak, H. E., Memisoglu, T., Gercek, Y., (2020)	GIS and AHP	Optimal site selection for solar photovoltaic (PV) power plants using GIS and AHP: A case study of Malatya Province, Turkey.
[59]	Castro-Santos, L., Garcia, G. P., Simões, T., Estanqueiro, A., (2019)	GIS	Planning of the installation of offshore renewable energies: A GIS approach of the Portuguese roadmap.
[60]	Asakereh, A., Soleymani, M., & Sheikhdavoodi, M. J. (2017).	GIS-based Fuzzy-AHP	A GIS-based Fuzzy-AHP method for the evaluation of solar farms locations: Case study in Khuzestan province, Iran.
[61]	Singh R, Pathak V.K., Kumar R., Dikshit M., Aherwar A., Singh V., Singh T. (2024)	Multi-objective optimization by ratio analysis” (MOORA) method and its fuzzy extensions	A historical review and analysis on MOORA and its fuzzy extensions for different applications.
[62]	Yasin K. H., Woldemariam G.W. (2023)	Geographic Information System (GIS)-based Multi-Criteria Decision-Making (MCDM) and Analytical Hierarchy Process (AHP)	GIS-based ecotourism potentiality mapping in the East Hararghe Zone, Ethiopia.

#### 2.2.1 Interval Type-2 fuzzy sets

Zadeh introduced first type-1 fuzzy sets and then type-2 fuzzy sets which is able to cope with uncertainty better than type-1 fuzzy sets. To reduce the computational complexity of type-2 fuzzy sets, interval type-2 fuzzy sets (IT2FSs) are proposed and the most researchers have preferred to use it in varieties of areas [63]. Therefore, in this study, interval type-2 fuzzy sets are used to solve the green helicopters selection problem.

Membership function characterized a fuzzy set namely $\tilde{A\;}$ can be shown as $\mu_{\tilde{A}}\left(x,u\right)$ and this fuzzy sets are depicted in equation (1):

(1) $$\tilde{A}=\{\left(x,u\right),{\;\mu }_{\tilde{A}}\left(x,u\right)|\forall x\in X,\forall u\in {\;J}_{x}\subseteq \left[0;1\right]\}$$

where each x in X and $u\in {\;J}_{x}\subseteq \left[0;1\right]$ where ${0\leq \mu }_{\tilde{A}}\left(x,u\right)\leq 1$. Interval type-2 fuzzy sets can be revealed where ${\;\mu }_{\tilde{A}}\left(x,u\right)=1$ in a fuzzy set $\tilde{A\;}$ [64]. Membership functions also can be defined in different shapes to accommodate uncertainty. These are triangular, trapezoidal, Gaussian and so on. In this study, trapezoidal interval type-2 fuzzy sets are used and can be depicted as equation (2):

(2) $${\tilde{A}}_{i}=\left({\tilde{A}}_{i}^{U},{\tilde{A}}_{i}^{L}\right)=\left(\left({\tilde{a}}_{i1}^{u},{\tilde{a}}_{i2}^{u},{\tilde{a}}_{i3}^{u},{\tilde{a}}_{i4}^{u};h_{1}\left({\tilde{A}}_{i}^{U}\right),h_{2}\left({\tilde{A}}_{i}^{U}\right)\right),\left({\tilde{a}}_{i1}^{l},{\tilde{a}}_{i2}^{l},{\tilde{a}}_{i3}^{l},{\tilde{a}}_{i4}^{l};h_{1}\left({\tilde{A}}_{i}^{L}\right),h_{2}\left({\tilde{A}}_{i}^{L}\right)\right)\right)$$

where ${\tilde{A}}_{i}$ comprises of two type-1 fuzzy sets; ${\tilde{A}}_{i}^{U}$ and ${\tilde{A}}_{i}^{L}$ where the reference points of ${\tilde{A}}_{i}$ are represented by ${\tilde{a}}_{i1}^{u},{\tilde{a}}_{i2}^{u},{\tilde{a}}_{i3}^{u},{\tilde{a}}_{i4}^{u},{\tilde{a}}_{i1}^{l},{\tilde{a}}_{i2}^{l},{\tilde{a}}_{i3}^{l}$ and ${\tilde{a}}_{i4}^{l}$ [49]. $h_{k}\left({\tilde{A}}_{i}^{U}\right)\;\text{and}\;h_{k}\left({\tilde{A}}_{i}^{U}\right)$ represent the height of each constituent membership function where 1≤k≤2. For this study, the height of each membership function is equal to 1 in IT2FSs and they are not considered during solution of the problem.

In addition, addition and multiplication algebraic operations are used in this study and two trapezoidal interval type-2 fuzzy sets ${\tilde{A}}_{1}$ and ${\tilde{A}}_{2}$ are used to show these algebraic operations in equation (3) and equation (4).

(3) $${\tilde{A}}_{1}=\left({\tilde{A}}_{1}^{U},{\tilde{A}}_{1}^{L}\right)=\left(\left({\tilde{a}}_{11}^{u},{\tilde{a}}_{12}^{u},{\tilde{a}}_{13}^{u},{\tilde{a}}_{14}^{u};h_{1}\left({\tilde{A}}_{1}^{U}\right),h_{2}\left({\tilde{A}}_{1}^{U}\right)\right),\left({\tilde{a}}_{11}^{l},{\tilde{a}}_{12}^{l},{\tilde{a}}_{13}^{l},{\tilde{a}}_{14}^{l};h_{1}\left({\tilde{A}}_{1}^{L}\right),h_{2}\left({\tilde{A}}_{1}^{L}\right)\right)\right)$$

and

(4) $${\tilde{A}}_{2}=\left({\tilde{A}}_{2}^{U},{\tilde{A}}_{2}^{L}\right)=\left(\left({\tilde{a}}_{21}^{u},{\tilde{a}}_{22}^{u},{\tilde{a}}_{23}^{u},{\tilde{a}}_{24}^{u};h_{1}\left({\tilde{A}}_{2}^{U}\right),h_{2}\left({\tilde{A}}_{2}^{U}\right)\right),\left({\tilde{a}}_{21}^{l},{\tilde{a}}_{22}^{l},{\tilde{a}}_{23}^{l},{\tilde{a}}_{24}^{l};h_{1}\left({\tilde{A}}_{2}^{L}\right),h_{2}\left({\tilde{A}}_{2}^{L}\right)\right)\right)$$

The addition operation symbolized by ⊕ in equation (5).

(5) $${\tilde{A}}_{1}⊕{\tilde{A}}_{2}=\left({\tilde{A}}_{1}^{U},{\tilde{A}}_{1}^{L}\right)⊕\left({\tilde{A}}_{2}^{U},{\tilde{A}}_{2}^{L}\right)=\left(\left({\tilde{a}}_{11}^{u}+{\tilde{a}}_{21}^{u},{\tilde{a}}_{12}^{u}+{\tilde{a}}_{22}^{u},{\;\tilde{a}}_{13}^{u}+{\tilde{a}}_{23}^{u},{\tilde{a}}_{14}^{u}+{\tilde{a}}_{24}^{u};\;\min\left(h_{1}\left({\tilde{A}}_{1}^{U}\right),h_{1}\left({\tilde{A}}_{2}^{U}\right)\right),\min\left(h_{2}\left({\tilde{A}}_{1}^{U}\right),h_{2}\left({\tilde{A}}_{2}^{U}\right)\right)\right),\left({\tilde{a}}_{11}^{l}+{\tilde{a}}_{21}^{l},{\tilde{a}}_{12}^{l}+{\tilde{a}}_{22}^{l},{\tilde{a}}_{13}^{l}+{\tilde{a}}_{23}^{l},{\tilde{a}}_{14}^{l}+{\tilde{a}}_{24}^{l};\min\left(h_{1}\left({\tilde{A}}_{1}^{L}\right),h_{1}\left({\tilde{A}}_{2}^{L}\right)\right),\min\;\left(h_{2}\left({\tilde{A}}_{1}^{L}\right),h_{2}\left({\tilde{A}}_{2}^{L}\right)\right)\right)\right)$$

The multiplication operation symbolized by ⊗ in equation (6).

(6) $${\tilde{A}}_{1}⊗{\tilde{A}}_{2}=\left({\tilde{A}}_{1}^{U},{\tilde{A}}_{1}^{L}\right)⊗\left({\tilde{A}}_{2}^{U},{\tilde{A}}_{2}^{L}\right)=\left(\left({\tilde{a}}_{11}^{u}\times {\tilde{a}}_{21}^{u},{\tilde{a}}_{12}^{u}\times {\tilde{a}}_{22}^{u},{\;\tilde{a}}_{13}^{u}\times {\tilde{a}}_{23}^{u},{\tilde{a}}_{14}^{u}\times {\tilde{a}}_{24}^{u};\;\min\left(h_{1}\left({\tilde{A}}_{1}^{U}\right),h_{1}\left({\tilde{A}}_{2}^{U}\right)\right),\min\left(h_{2}\left({\tilde{A}}_{1}^{U}\right),h_{2}\left({\tilde{A}}_{2}^{U}\right)\right)\right),\left({\tilde{a}}_{11}^{l}\times {\tilde{a}}_{21}^{l},{\tilde{a}}_{12}^{l}\times {\tilde{a}}_{22}^{l},{\tilde{a}}_{13}^{l}\times {\tilde{a}}_{23}^{l},{\tilde{a}}_{14}^{l}\times {\tilde{a}}_{24}^{l};\min\left(h_{1}\left({\tilde{A}}_{1}^{L}\right),h_{1}\left({\tilde{A}}_{2}^{L}\right)\right),\min\;\left(h_{2}\left({\tilde{A}}_{1}^{L}\right),h_{2}\left({\tilde{A}}_{2}^{L}\right)\right)\right)\right)$$

#### 2.2.2 Taguchi loss function

Taguchi loss function introduced by Taguchi is a way to calculate the loss considering a certain level of quality during production process of a company. Based on the characteristic of the quality, there are three assumptions to form Taguchi loss function; a) nominal is better, b) lower is better, and c) larger is better. To minimize the variation between the target and actual values, a quadratic function is used. In this study, the characteristic of the quality is assumed that larger is better and the loss function is measured in equation (7):

(7) $$L\left(m\right)=K*m^{2}$$

where L(m) depicts the loss revealed during the production process for an actual measurement m along with a loss coefficient K and this loss coefficient can be shown in equation (8):

(8) $$K=100\;\%÷U^{2}$$

where U represents the upper specification limit for each m [65].

## 3 Material and methods

**Fig. 1:**
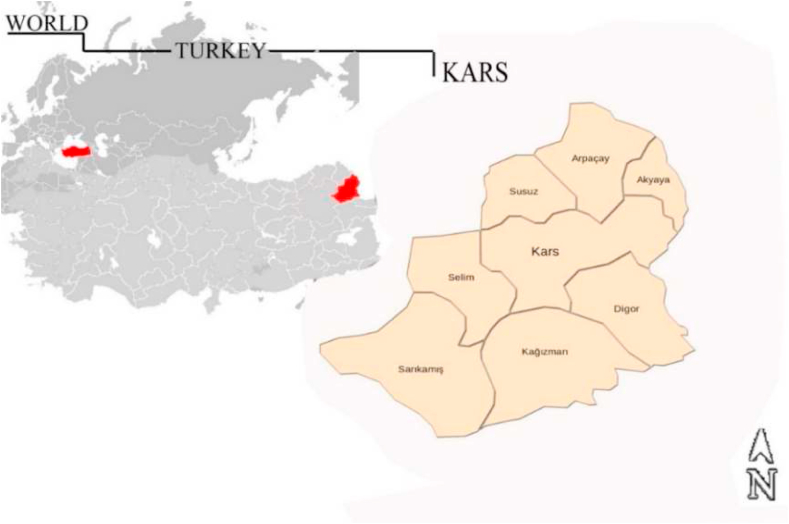
Kars province map.

In terms of Turkey's solar potential, the Eastern Anatolia Region is considered to be the 3rd place among 7 regions with 2664 h of sunshine duration and 1365 kWh/m^2^ annual radiation value. Kars Province (Fig. 1), which is located in the Eastern Anatolia Region with a surface area of approximately 10,127 km^2^, is topographically composed of undulating plains with river valleys and plateaus. In terms of its surface area being composed of such wide plains, it allows the establishment of many solar power plant fields in Kars on a provincial scale.

The aim of this study is to examine the 8 disrict regions of the Kars province - K1(Arpacay), K2 (Susuz), K3 (Akyaka), K4 (Kars Centrial), K5 (Digor), K6 Kagizman, K7 Sarikamis and K8 Selim.- for the establishment of solar power plant in the Kars/Turkey. The alternatives were evaluated according to various criteria such as (C1) ground surface temperature, (C2) electricity transmission, (C3) air pressure, (C4) air humidity, (C5) wind speed, (C6) air temperature, (C7) land use, (C8) solar radiation, (C9) aspect, (C10) slope, (C11) transport network, (C12) elevation layers using Arc-GIS mapping technique and Multi-Criteria Decision Making (MCDM), one of the Taguchi loss function based interval type-2 fuzzy methods. Also it is aimed to create a provincial-scale solar energy potential map, taking into account the ecological risks and ecological criteria, in a way that will ensure the least damage to the natural balance of Kars province.

### 3.1 Criteria for solar power plant

We have determined some criteria for the solar power plant site selection problem in Kars province in line with the existing literature and expert opinion. The criteria used in this study are as follows.

#### 3.1.1 Slope

**Fig. 2:**
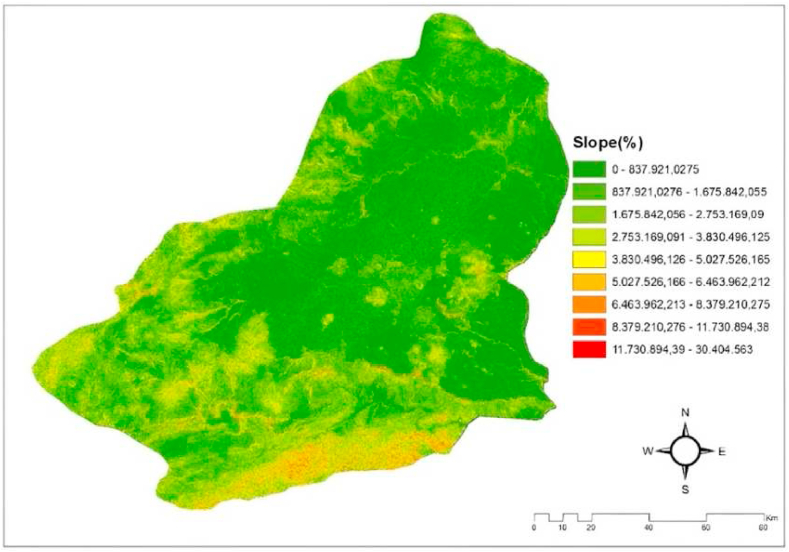
The slope map of the Kars province/Turkey [19,20,32].

The ratio of the horizontal displacement between two places to the vertical elevation difference. Elevation refers to the vertical difference between two places at a specified horizontal distance. The height difference is utilized to ascertain the angle of incidence of solar energy in solar energy installation. The slope map of Kars province is shown in Fig. 2 [19,20,32].

#### 3.1.2 Aspect

**Fig. 3:**
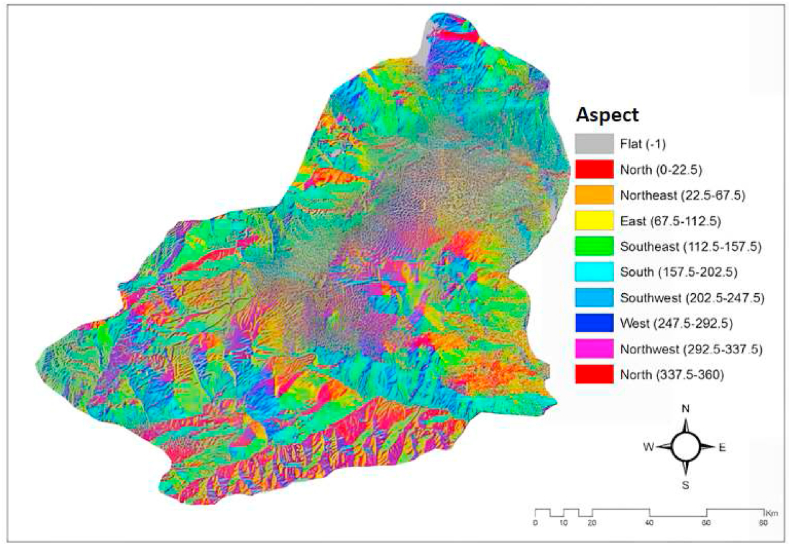
The aspect map of the Kars province/Turkey [19,20,32].

It can be explained as the direction of reception of the sun's rays or the part of the sun in a region. It occurs as a result of the mathematical position due to the effect of latitude. The aspect maps were created in the Spatial analyst module of the Arc-GIS 10.2 package program and classified in 8 different categories to indicate the directions. The aspect map of Kars province is shown in Fig. 3 [19,20,32].

#### 3.1.3 Solar irradiation

**Fig. 4:**
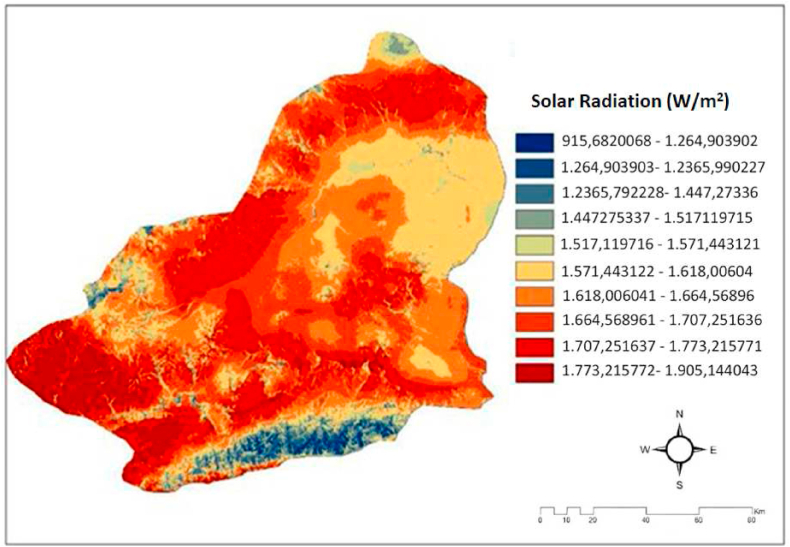
The map for solar irradiance in Kars province [19,20,32].

Solar radiation refers to the electromagnetic radiation emitted by the Sun, including visible light, ultraviolet (UV) radiation, and infrared (IR) radiation. The Sun emits various forms of electromagnetic radiation, including light, heat, and other types of waves. The Sun is the primary and most significant provider of energy on Earth. The most compelling evidence of this is the phenomenon that, despite its vast distance spanning millions of kilometers, the sun is able to effectively warm the earth and supply it with energy. Sunlight is the primary catalyst for energy transfer in ecosystems. In the field of solar energy, the incident sunlight on the solar panel induces conductivity in the semiconductor materials, thereby initiating the process of energy production. The panel is illuminated by sunshine, serving as an energy source for the semiconductor material. At this point, the semiconductor transitions into a conductor, initiating the production of electrical energy. The process involved accessing the Solar Radiation button in the Patial analyst module of the Arc-GIS 10.2 package program and selecting the Area solar radiation tab to generate the desired results from the DEM maps.In Fig. 4, we can see the solar radiation and solar power plants map for Kars province [19,20,32].

#### 3.1.4 Wind speed

**Fig. 5:**
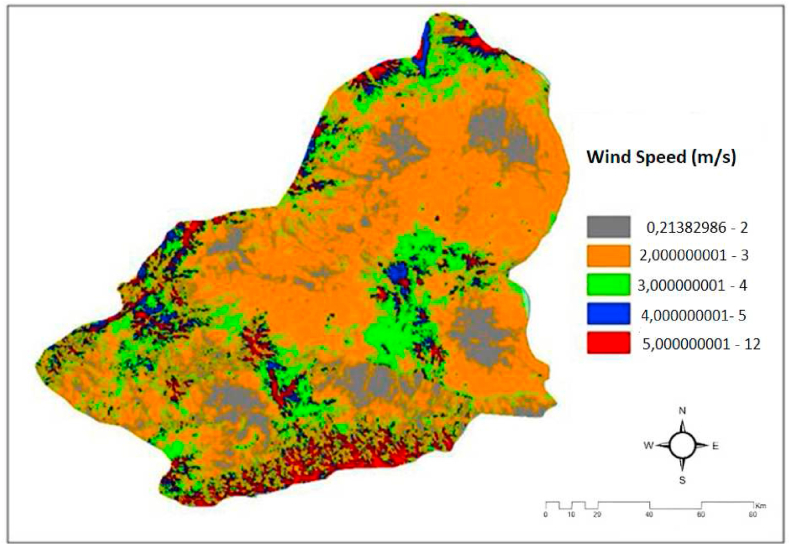
The map for Wind speed in the Kars province/Turkey [19,20,32].

Wind is the horizontal movement of high-pressure air towards low-pressure areas. This displacement of air is called wind. The main cause of wind formation is temperature. Regional temperature differences cause the wind to move. As the temperature increases, the atoms in the molecular structure of the air move faster and their energy levels increase. In addition to being a renewable energy source, it has a serious potential in stabilizing the air temperature. Wind energy turbines are used in energy production. DEM maps were created by selecting the Area solar radiation tab in the Solar Radiation button in the Patial analyst module of the Arc-GIS 10.2 package program. Fig. 5 shows the wind speed map in Kars province [[66], [67], [68]].

#### 3.1.5 Air temperature

**Fig. 6:**
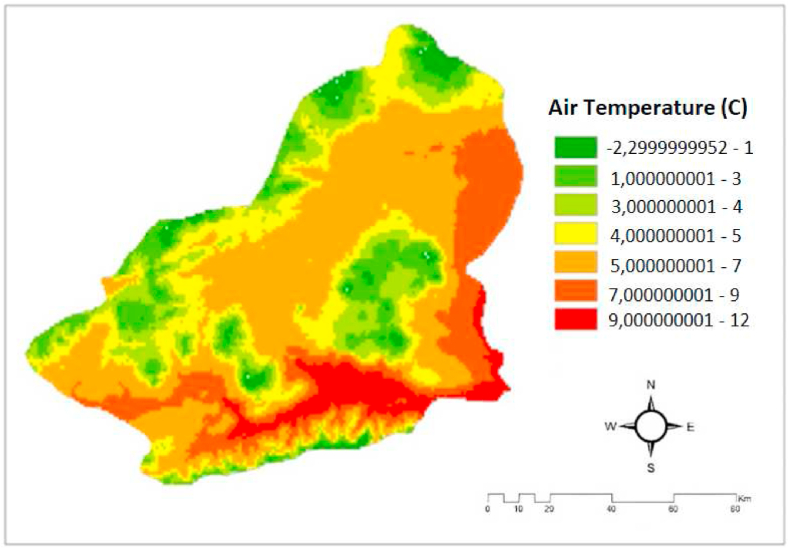
The map for Air Temperature in the Kars province/Turkey [19,20,32].

Air Temperature: Air temperature is the result of the oscillations of gas molecules in the atmosphere due to the presence of thermal energy. The primary determinant of climate is the presence of moisture, precipitation, pressure, and wind, among other factors. Temperature is the measure of the average kinetic energy of the particles constituting a substance. Particles in matter transmit their kinetic energy through collisions, resulting in continual changes in the kinetic energy of each particle. Given that matter particles possess varying kinetic energies at a given temperature, it is important to note that the temperature represents the average kinetic energy of all particles, rather than that of an individual particle. Studies have shown that energy output and efficiency are enhanced by reducing panel temperature through air circulation in hot areas exposed to sunlight, whereas energy production and efficiency decline when air circulation is present. The air temperature maps of the study area were digitally acquired from the "Global Atlas". They were then reclassified using the Reclass button in the Spatial Analyst Tool module in the Arc-GIS 10.2 package application. This reclassification was done to produce a structure that is consistent with the scale of the study. The temperature map of Kars province is shown in Fig. 6 [19,20,32].

#### 3.1.6 Land use

**Fig. 7:**
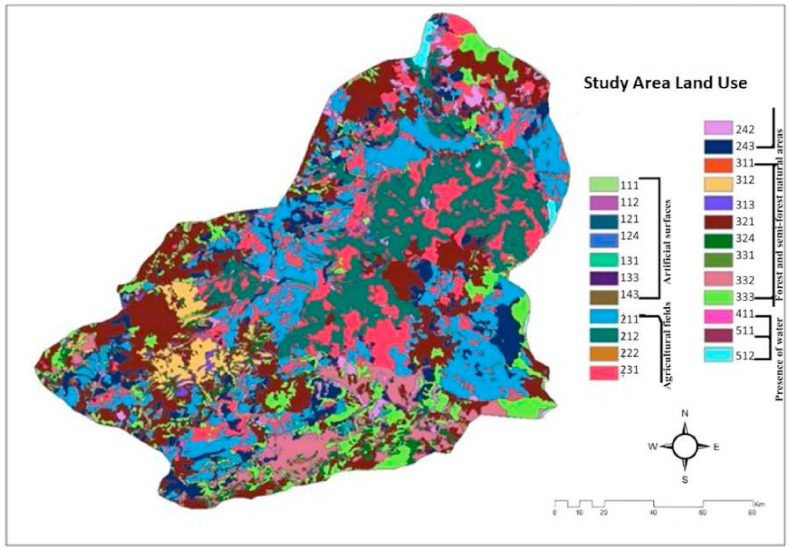
The map for Land Use in the Kars province/Turkey [19,20,32].

Land use refers to the governance and control of how land within a country or region is utilized, safeguarded, and administered. The objective of land use is to safeguard the economic, social, and environmental concerns of land in a harmonized manner. Land use include the safeguarding of the land's natural resources, land use planning, agriculture, forestry, tourism, energy generation, transportation, identification of locations suitable for renewable energy, and other economic activities aligned with land use. The purpose of land use is to optimize the utilization of land resources in response to the growing population, while also safeguarding the integrity of the natural environment, establishing livable residential areas, preserving water basins and wetlands, and developing technical infrastructure zones. The land use map of Kars province is shown in Fig. 7 [19,20,32].

#### 3.1.7 Transmission line

**Fig. 8:**
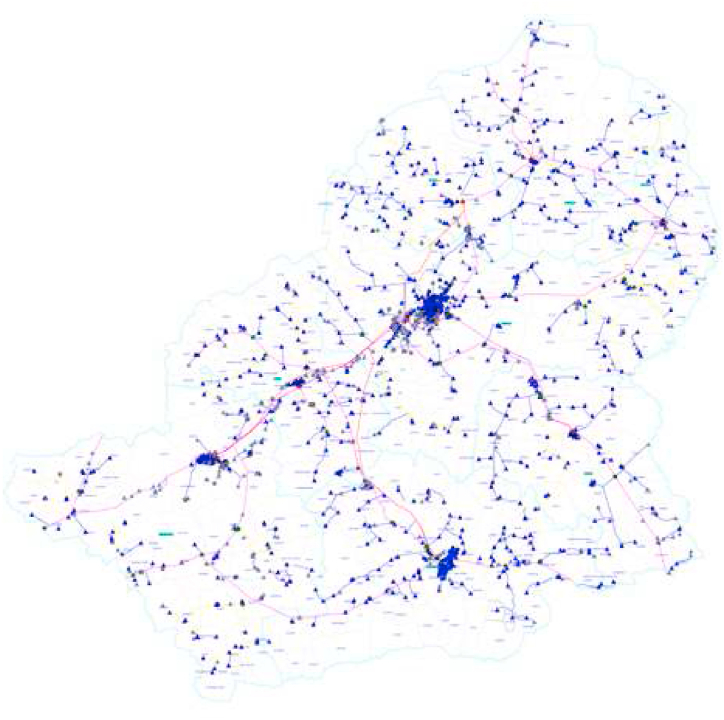
The transmission lines in the Kars province [19,20,32,69].

It is a system that facilitates the transmission of electrical energy from power plants to transformer stations located near regions where electricity is consumed. When laying electricity lines, various factors are considered, including cost, transmission line route, geographical and land conditions, operational efficiency, voltage drop calculation, and capacity requirements. Ensuring the safe construction of the power line and minimizing electricity losses during transmission is of utmost importance. Fig. 8 displays the transmission line maps for the Erzurum province. The raster data for the Electricity Distribution map was digitized using the ArcGIS 10.2 software package. The digitized data were analyzed using the Buffer Analysis application [19,20,32].

#### 3.1.8 Air humidity

Humidity refers to the water vapor that is transported by the air. The air's capacity to hold water vapor, known as moisture, fluctuates in accordance with temperature and pressure. Raising the temperature leads to an increase in the capacity to hold moisture. As humidity levels rise, the perceived temperature drops. Lowering the temperature also reduces the interior temperature of the panel, resulting in an improvement in energy efficiency. The humidity maps of the research area were created by converting the point data collected from stations in 12 distinct locations into digital format using the Arc-GIS 10.2 software package. The "Kriging" method in the Geostatistical Analyst module was then applied to generate the maps [70].

#### 3.1.9 Air pressure

The atmosphere of the Earth possesses mass and exerts gravitational force. Air pressure refers to the gravitational force exerted by the gases comprising the Earth's atmosphere. The pressure interacts with humidity and temperature to generate wind. Wind generation results in a drop in the temperature of the panel. A reduction in the interior temperature of the panel leads to an improvement in energy efficiency. The Air Pressure map was digitized using the Arc-GIS 10.2 software package and obtained by the application of the "Kriging" method in the Geostatistical Analyst module [[70], [71], [72]].

#### 3.1.10 Land surface temperature

Land surface temperatures have a crucial role in climate and are utilized in climate models. It is effectively employed to ascertain the values and variations of land surface temperature (LST) and to examine their impacts [73]. Land Surface Temperature varies based on land cover and land use, and is commonly employed to assess changes in the land. In several studies such as urban heat island, climate, agriculture [74], drought, forestry, and maritime, among others, data on land surface temperature (LST) is considered a crucial and efficient source of information [[75], [76], [77], [78]]. To accurately compute the LST value, it is necessary to have data on atmospheric effects values, sensor parameters, and ground surface emissivity. These data are essential for the computation of LMS [79]. The calculation of Land SurfaceTemperature (LST) utilizes many approaches including Single-channel, Multi-angle, Multi-channel, and Split Window techniques [80]. In the single channel technique, the variation in radiation emitted from the atmosphere and recorded on Earth is directly proportional to the data observed in two distinct channels. By employing this method, one can achieve atmospheric effect and surface radiation values [84]. The thermal satellite pictures include data on atmospheric temperature, humidity, water vapor, and brightness temperature [81]. Land surface temperatures ranging from −39 °C to 30 °C were analyzed in the study area. Kars province has a continental climate. Summers are dry and winters are rainy, and temperatures drop to −39 °C in winter. The number of snow-covered days is more than 120. The climate type in this province differs from other eastern provinces. For example, it can be shown that the most precipitation falls in the summer season. Within the province of Kars, temperature differences are observed from district to district in the summer months. For example, Akyaka, Arpaçay, Digor and Kağızman districts are hotter than Sarıkamıș, Selim and Susuz, especially the central district.

**Fig. 9:**
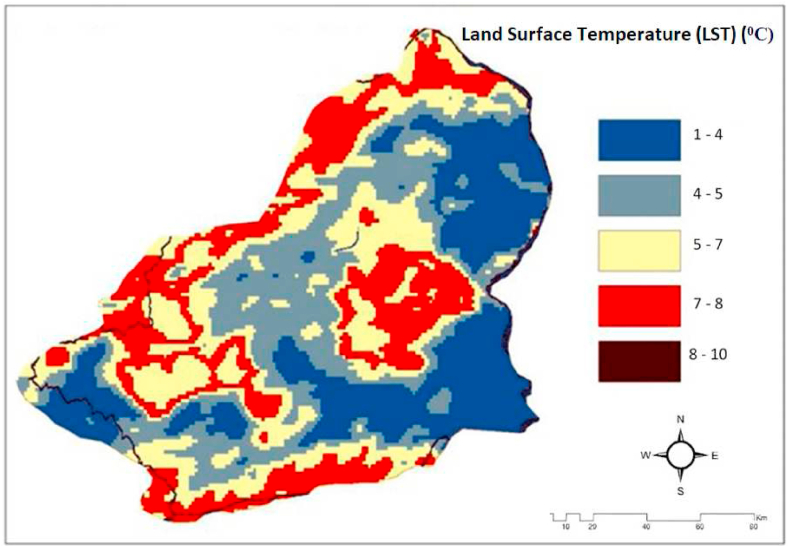
Land surface temperatures map of the Kars Province [24,39,69].

**Fig. 10:**
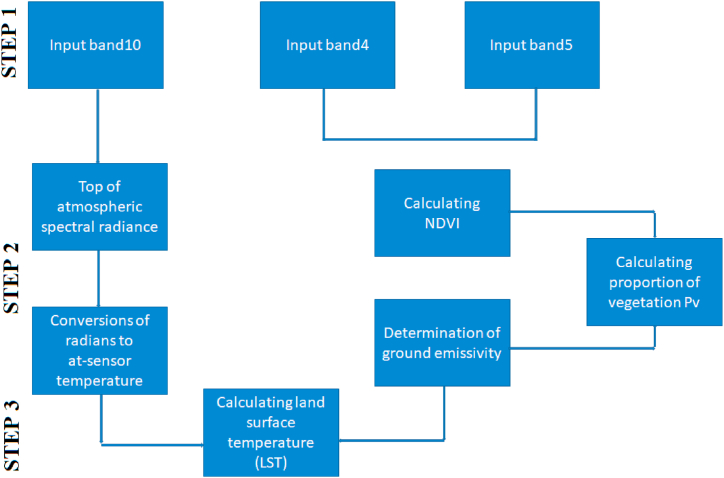
Flowchart showing the stages of creating LST maps of the study area [20].

Regions with significant rainfall are characterised by low surface temperatures. This is an undesirable scenario for the utilization of solar energy. This leads to a decrease in the frictional force near the ground as well as a decrease in wind intensity and thus in the Coriolis force. This phenomenon reduces the overall movement of the atmosphere and wind generation [19,33,82,83]. Fig. 9 shows the land surface temperature. The procedure for obtaining the LST is shown in Fig. 10.

The band data from the Landsat 8 satellite was analyzed using ArcGIS 10.8 software, as shown in the diagram above [19,20,33,[84], [85], [86]]. During the initial phase of the investigation, the satellite images captured by Landsat 8 were analyzed and classified according to different bands. The atmospheric radiation value in this stage was determined using the following equation.

Lλ is the spectral brightness at the sensor aperture and is calculated using the formula Band10x0.003342 + 0.1.

The unit of measurement for Lλ is watts per square meter per steradian per micrometer (W/(m 2 sr μm).

During the second stage, an additional equation was employed to translate the atmospheric irradiance value into the sensor temperature [87]. The formula to calculate TB is as follows:

TB = (1321.08 / Ln((774.89 / (Lλ)) + 1)) - 273.15

The following equation was employed to derive NDVI maps [77,87,88]. Normalized plant density index maps are crucial as they illustrate variations in temperature fluctuations of the natural surface based on plant densities [89,90].

The normalized difference vegetation index (NDVI) is calculated by dividing the difference between Band 5 and Band 4 by the sum of Band 5 and BandNDVI refers to maps that represent the normalized plant density index.

In the subsequent phase of the investigation, the computation of the vegetation ratio was conducted. The vegetation ratio was calculated using the algorithm provided [91]. Pv is alculated using the formula Pv = 0.004x(NDVI) + 0.986. Pv represents the vegetation ratio.

During the last phase of the investigation, highly accurate maps of surface temperature (LST) were generated with minimal margin of error.

#### 3.1.11 Altitude

**Fig. 11:**
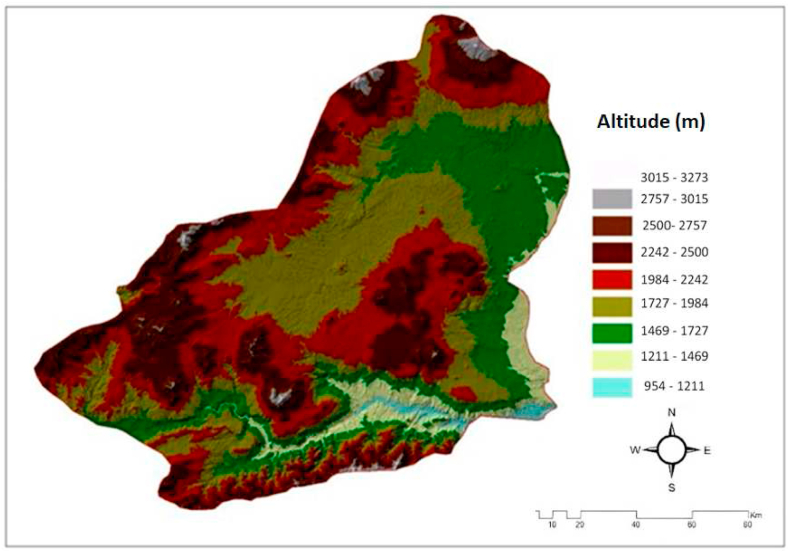
The altitude map of the Kars Province.

Altitude is the height of any object relative to a known level. Usually this known level is mean sea level. It is the average metre of the highest and lowest area. The same landforms can exist at different heights and different landforms can exist at the same heights. Altitude is related to wind speed. It is the vertical distance of a point on the earth from the sea. This is called the height or altitude of a place above sea level. Altitude improves performance wind power as a result of an increase in wind speed [92]. Turkey's elevation maps were created by digitising 1/250000 raster map sheets in the Formiturul Tool module of the Arc GIS10.2 Spatial Analyst Package programme of the General Command. These maps were converted into DEM format in the Conversion Tool module of Arc-GIS 10.2 package programme. The elevation map of Kars is shown in Fig. 11 [93].

## 4 Results

In this study, after a model is created in Arc-GIS 10.2 software, raster data sets and digital data are evaluated [13,40,41]. The data sequence and analysis in this model is shown in Fig. 14. Before creating the model, (C1) ground surface temperature, (C2) electricity transmission, (C3) air pressure, (C4) air humidity, (C5) wind speed, (C6) air temperature, (C7) land use, (C8) solar radiation, (C9) aspect, (C10) slope, (C11) transport network, (C12) elevation, respectively.

### 4.1 Applying GIS

**Fig. 12:**
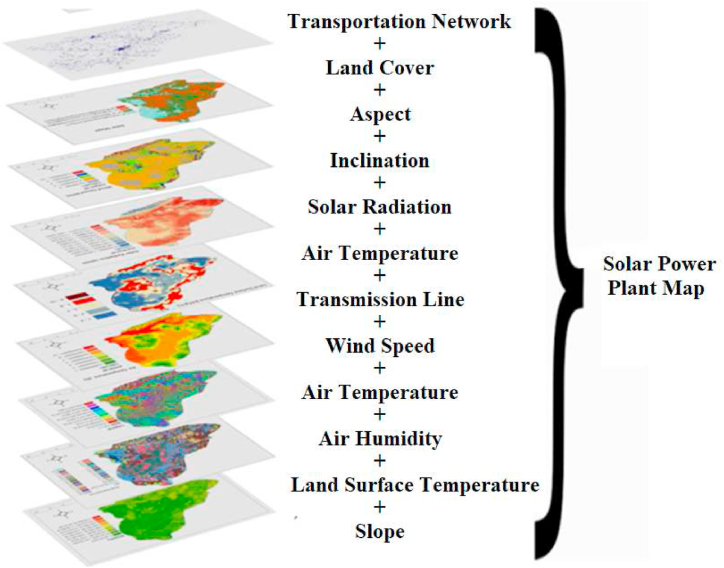
Model created for study result maps [19,20,33].

Map design is a multifaceted production process including map scale and purpose. It is the encoding of the real world for the summarisation of the earth's reality and its presentation by means of a map (Table 2). It is the design by selecting the primary spatial relations, objects and characteristic aspects of geographical reality for purposeful use. It is the selection of objects to be presented depending on the scale of the map and the degree of summarisation. In this process, layers expressing different characteristics of a place should be transferred to geographically referenced databases and converted into the same data format. The pixels forming each map in raster format are given numerical values. The registration process consisting of the multiplication of these numerical values is a mathematical operation and allows millions of operations to be performed simultaneously. As a result of this process, a result map, which is also a different map, is obtained from the analysis of layers with different properties (Fig. 12).

**Table 2:** Suitable location for Solar Power Plant Scoring values created by experts for each layer [20,82,93].

Evaluation factor for solar energy	Selected sub-units for solar energy	Relevance Number for solar energy
**Elevation**	697–1000 m	4
	1000–1500 m	1
	1500–2000 m	1
	2000–4257 m	1
**Slope**	Flat	4
	Slightly sloping	4
	Sloping	3
	Very Sloping	1
**Land Cover**	Industrial Commercial and Transports	1
	Mine and Construction Areas	1
	Non-Agricultural Artificial Green Areas	2
	Fields Suitable for Agriculture	1
	Continuous Products	1
	Pasture Area	3
	Heterogeneous Agricultural areas	1
	Forests	1
	Shrubbery Areas	2
	Areas Without Vegetation	3
	Interior Wet Areas	1
	Wet Areas Near the Shore	1
	Inland Waters	1
	Water surfaces	1
	Grassland	4
	Farmland	1
	Natural meadows	4
	Infrastructure of cities	1
**Aspect**	North	1
	Northeast	1
	East	2
	Suoutheast	2
	South	4
	Southwest	3
	West	1
	Northwest	1
**Inclination**	Flat (0–2%)	1
	Slightly inclined (2–6%)	1
	Middle (6–12 %)	1
	Steep (12–20 %)	1
	Very Steep (20–30 %)	1
	scarped 30+%)	1
**Solar Radiation**	Irradiation excess	4
	Irradiation too	3
	Irradiation normal	2
	Irradiation low	1
**Air Temperature**	Hot	2
	A Bit Hot	4
	Normal	4
	Cool	2
**(distance to) Transmission Line**	0–500 m	4
	500–1000 m	3
	1000–1500 m	2
	1500–2000 m	1
	2000 m>	1
**Wind Speed**	Hard Wind (10–12)	3
	Windy (8–10)	4
	Wind Light (5–8)	4
	No Wind or Low (0–5)	1
**Air Pressure**	High	4
	Normal	3
	Low	2
	Very Low	1
**Air Humidity**	High	1
	Normal	2
	Low	3
	Very Low	4
**Land Surface Temperature (LST)**	Very Hot	1
	Hot	2
	Normal	3
	Cool	4
**Transportation Network**	0–500 m	4
	500–1000 m	4
	1000–1500 m	3
	1500–2000 m	2
	2000 m>	1

The final result map was produced and the results were interpreted in two different ways; the first is the GIS method. In this method, the result map was obtained by giving scores between 1 and 4 according to the weighting given in Table 2. The second is the weights determined by the interval type-2 fuzzy-based approach based on the Taguchi loss function. Here again a different scoring system was used.

### 4.2 Applying taguchi loss function based interval type-2 fuzzy approach

**Fig. 13:**
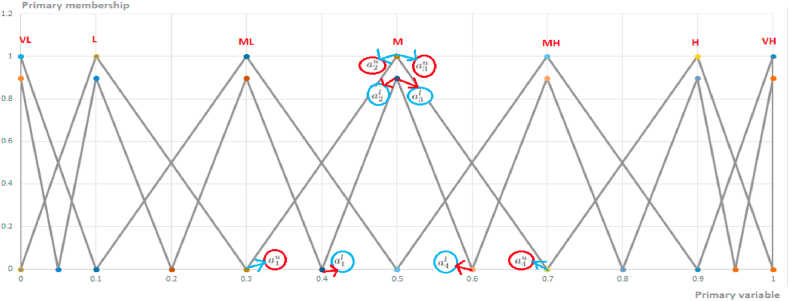
Illustration of IT2FSs for ′Medium' (M).

In this study, criteria were identified based on expert opinions and existing literature for the overall evaluation process of the potential solar energy fot 8 disrict in Kars, namely K1(Arpacay), K2 (Susuz), K3 (Akyaka), K4 (Kars Centrial), K5 (Digor), K6 Kagizman, K7 Sarikamis and K8 Selim. First, the importance of twelve criteria ((C1) land surface temperature, (C2) electrical transmission, (C3) air pressure, (C4) air humidity, (C5) wind speed, (C6) air temperature, (C7) land use, (C8) solar radiation, (C9) aspect, (C10) slope, (C11) transportation network, (C12) elevation) were rated and then the performance of each alternative was analyzed in terms of these criteria. First, the importance of twelve criteria is rated and then the performance of each alternative is examined in terms of these criteria. Linguistic terms ′Very Low' (VL), ′Low' (L), ′Medium Low' (ML), ′Medium' (M) (In Fig. 13), ′Medium High' (MH), ′High' (H), ′Very High' (VH) are used to determine the importance of criteria and their corresponding fuzzy sets are demonstrated in Table 3. For performance evaluation of each solar energy, the linguistic terms ‘Very Poor' (VP), ‘Poor' (P), ‘Medium Poor' (MP), ‘Fair' (F), ‘Medium Good' (MG), ‘Good' (G) and ‘Very Good' (VG) are used. The associated fuzzy sets for each term are shown in Table 4.

**Fig. 14:**
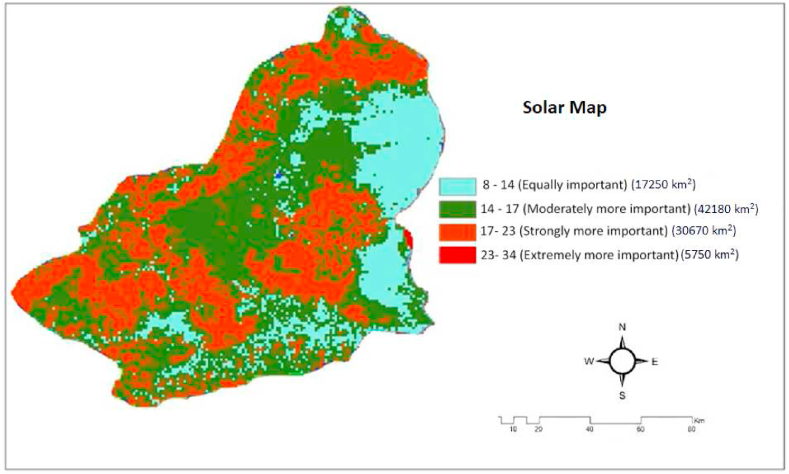
Kars province solar power plant installation map.

**Table 3:** Linguistic variables to represent the importance of criteria [94].

Trapezoidal ITFSs	$\alpha_{1}^{u}$	$\alpha_{1}^{u}$	$\alpha_{1}^{u}$	$\alpha_{1}^{u}$	$\alpha_{1}^{I}$	$\alpha_{1}^{I}$	$\alpha_{1}^{I}$	$\alpha_{1}^{I}$
Very Low (VL)	0	0	0	0.1	0	0	0	0.05
Low (L)	0	0.1	1.1	0.3	0.05	0.1	1.1	0.2
Medium Low (ML)	0.1	0.3	0.3	0.5	0.2	0.3	0.3	0.4
Medium (M)	0.3	0.5	0.5	0.7	0.4	0.5	0.5	0.6
Medium High (MH)	0.5	0.7	0.7	0.9	0.6	0.7	0.7	0.8
High (H)	0.7	0.9	0.9	1	0.8	0.9	0.9	0.95
Very High (VH)	0.9	1	1	1	0.95	1	1	1

**Table 4:** Linguistic variables to represent the performance of alternatives [94].

Trapezoidal ITFSs	$\alpha_{1}^{u}$	$\alpha_{1}^{u}$	$\alpha_{1}^{u}$	$\alpha_{1}^{u}$	$\alpha_{1}^{I}$	$\alpha_{1}^{I}$	$\alpha_{1}^{I}$	$\alpha_{1}^{I}$
Very Poor (VP)	0	0	0	1	0	0	0	0.5
Poor (P)	0	1	1	3	0.5	1	1	2
Medium Poor (MP)	1	3	3	5	2	3	3	4
Medium (M)	3	5	5	7	4	5	5	6
Medium Good(MG)	5	7	7	9	6	7	7	8
Good (G)	7	9	9	10	8	9	9	9.5
Very Good (VG)	9	10	10	10	9.5	10	10	10

The explanation of this approach can be done in six steps as follows.

**Step 1:** Decision maker determined six criteria as detailed in Section 2. The importance of each criterion is decided by the decision-maker as shown in Table 5.

**Table 5:** Importance of criteria determined by a decision maker.

Abb.	Criteria					
	C1	C2	C3	C4	C5	C6
	VH	M	VH	ML	H	VH
**Step 2:** The performance of each alternative is assigned using linguistic terms in terms of six criteria for each state as seen in Table 5,
**Step 3:** Linguistic terms are converted into fuzzy numbers shown in Table 4 for importance of criteria and Table 4 for performance of alternatives.
**Step 4:** Fuzzy sets for the performance of alternatives are multiplied by their associated fuzzy sets for the importance of criteria. For a better understanding, let us compute the importance of C1signed as “VH” and the performance of A1 assigned as “VH” for the first state:

(9) C1(VH)⊗A1(VH)=((0.9,1,1,1),(0.95,1,1,1))⊗((9,10,10,10),(9.5,10,10,10))=((8.1,10,10,10,),(9.025,10,10,10))

in the same manner, all alternatives and criteria are computed for five states.

**Step 5:** These fuzzy sets obtained in the previous step are converted into crisp values using Centroid type-reduction and defuzzification methods. In the proposed approach, Taguchi loss function is used as an alternative type-reduction and defuzzification method.
**Step 6:** After type-reduction and defuzzification process, we achieve crisp values and alternatives are ranked according to crisp values in Table 6.

**Table 6:** Importance of criteria according to the decision-maker for Solar power plant.

	C1	C2	C3	C4	C5	C6	C7	C8	C9	C10	C11	C12
**Decision Maker**	I	I	I	VI	VI	VI	VI	I	I	VI	VI	VI

As shown in Table 7, pairwise comparison matrices for five application areas have been created and shown.

**Table 7:** Performance of alternatives according to the decision-maker for Solar power plant.

Alternatives	C1	C2	C3	C4	C5	C6	C7	C8	C9	C10	C11	C12
K1	EH	ML	H	EH	EH	EH	EH	MH	M	ML	VH	EH
K2	ML	M	MH	H	MH	EL	EL	M	H	MH	H	MH
K3	EH	EH	H	M	M	H	M	M	MH	VH	MH	VH
K4	ML	M	MH	H	MH	EL	EL	M	H	MH	H	MH
K5	EH	EH	VH	ML	L	VH	M	ML	H	H	H	H
K6	ML	M	H	VH	H	M	L	M	EL	H	EL	H
K7	EH	ML	H	EH	EH	EH	EH	MH	M	ML	VH	EH
K8	EH	EH	VH	ML	L	VH	M	ML	H	H	H	H

Table 7 shows the performance of each of the alternatives respectively according to the decision maker for the solar power plant. Decision makers rank the scores according to the importance of each criterion as in Table 8 for the Solar Power Plant.

**Table 8:** For solar power plant positive and negative distances, closeness coefficients and the rank of alternatives.

Alternatives	Positive-ideal	Negative-ideal	Closeness Coefficients	Rank
K1(Arpacay)	0.4005	0.1910	0.3013	4
K2 (Susuz)	0.3439	0.1533	0.2346	5
K3 (Akyaka)	0.1866	0.3533	0.1153	7
K4 (Kars Centrial)	0.2402	0.2136	0.1399	6
K5 (Digor)	0.2918	0.1213	0.2914	5
K6 Kagizman	0.1514	0.3428	0.3915	1
K7 Sarikamis	0.3318	0.1136	0.3895	2
K8 Selim	0.3767	0.2627	0.3532	3

According to this result, it was determined that Kağızman (K6) is the best place to establish a solar power plant among 8 districts. According to the net score we obtained, the areas suitable for solar energy installation are listed in Table 8. As an experimental result, the result obtained with GIS is shown in Fig. 14. According to the obtained solar power plant map, 5750 km2 area is the most important area for power plant installation, 30670 km2 area is the most important area for power plant installation, 42180 km2 area is the most suitable area for power plant installation and 17250 km2 area is the most unsuitable area for power plant installation. In total, 78600 km2 is the area that can be used for power plant installation. We obtain approximately 78,600 mWh of solar energy from this area. In Fig. 14, we see our last map, the solar energy installation map.

## 5 Discussion

As a result of the literature review, we can see only the study of [95] on solar energy site selection in Kars province. In Ref. [95]'s study, the results obtained in this experimental study using the Analytical Hierarchy Process (AHP) method for determining the lands with potential suitable for solar power plant (SPP) installation in Kars province overlap to a great extent with the installation locations of solar power plants that are still in active production by making application projects throughout the province. This confirms the reliability and validity of the research. When we look at Fig. 14 in our study, we see that Kars Province is generally suitable for the installation of solar power plants on flat areas. In this study, GIS - Taguchi loss function based interval type-2 fuzzy approach is used for the first time [95]. has used 7 parameters in this study and we have done a more in-depth research by using 12 criteria for solar power plant installation for the first time in this study. In [96]'s study, 7 environmental parameters were examined and we determined that 9860 km^2^ area is suitable for solar power plant. When we examined our 12 environmental parameters in detail, we determined that 78600 km^2^ area is suitable for solar power plant. Despite this unbalanced and inconsistent situation in terms of the weights and geographical distribution of the criteria used in the research, the fact that the installation locations of the solar power plants in the province are in parallel with the areas determined in this research is generally in line with the solar power plant map we obtained in Fig. 14 with the Taguchi loss function based interval type-2 fuzzy approach used in the research. This shows the reliability and validity of the results. Kars has a continental climate. The climate of the highlands of Kars, which belongs to the Kars region, is quite harsh due to the fact that it is high and separated from the sea by mountain ranges. In the province where winters are dry and summers are rainy, temperatures drop to −39 °C from time to time in winter, the driest season. The average number of snow-covered days is over 120. This climatic condition is important for the installation of a solar power plant. According to the results of our study, we see that Kağızman province has the best areas in terms of solar energy. In this study, the environmental parameters affecting the efficiency of a photovoltaic solar power system are analyzed and the measures to be taken to keep the performance high are discussed. We have analyzed the environmental parameters in more detail than previous studies. In this way, this article is intended to provide a basis for research on solar power plant site selection in Kars province and for investors who want to establish a solar power plant.

## 6 Conclusion

In this study, in addition to some criteria in the literature, twelve criteria that are not included in the literature but are necessary for a solar power plant were used to determine the most suitable location for a solar power plant. These 12 criteria are slope, aspect, elevation, solar radiation, land use, wind speed, air temperature, air pressure, humidity, land surface temperature and electricity transmission line. The significance values of the proposed new criteria and all sub-criteria were found using GIS method. We addressed the problem of locating the most suitable solar power plant by considering both qualitative and quantitative factors in 8 districts of Kars province. We solved this problem in two ways: Using GIS mapping technique and using Taguchi loss function based interval type-2 Fuzzy approach. The results of these two applications, namely the GIS mapping technique and the heuristic fuzzy-based approach, were compared and verified. As a result of the solar energy map obtained as a result of these two approaches and the annual data obtained, it was determined that Kağızman District showed high solar energy potential. Sarıkamıș district came second. The settlements in Kars province show a homogenous distribution. Due to this homogenous distribution, there is no restriction on the distribution of energy transmission lines in the site selection of solar power plants to be established in Kars province. Most of the marginal lands where solar power plants can be established consist of sloping land. Therefore, the cost of solar power plant installation increases. According to the obtained solar power plant map, 5750 km^2^ area is the most important area for power plant installation, 30670 km^2^ area is the most important area for power plant installation, 42180 km^2^ area is the most suitable area for power plant installation and 17250 km^2^ area is the most unsuitable area for power plant installation. In total, 78600 km^2^ is the area that can be used for power plant installation. We obtain approximately 78,600 mWh of solar energy from this area.

## Description

This article was written by İbrahim Akkuş in Iğdır University, Institute of Science and Technology under the supervision of Assoc. Prof. Gökhan Şahin. It is derived from Ibrahim Akkus's master thesis entitled (Site Selection of Solar Power Plants in Kars Province and Effects of Environmental Factors on Solar Energy Efficiency).

## Ethical approval and permission to participate

This article does not require ethical approval or permission to participate.

## Release permission

The authors performed this work themselve. There was no need for a release from elsewhere.

## Availability of data and material

The data used in the publication were obtained from meteorology data made available by KNMI.

## Financing

The work in this paper is performed without funding sources.

## CRediT authorship contribution statement

**Gokhan Sahin:** Writing – review & editing, Supervision, Software, Methodology, Investigation. **İbrahım Akkus:** Writing – review & editing, Methodology, Investigation. **Ahmet Koc:** Writing – review & editing, Methodology. **Wilfried van Sark:** Writing – review & editing, Investigation.

## Declaration of competing interest

The authors declare the following financial interests/personal relationships which may be considered as potential competing interests:Gokhan Sahin reports financial support was provided by Faculty of Geosciences, Copernicus Institute of Sustainable Development, 10.13039/501100001829Utrecht University. Gokhan Sahin, Researcher, Geoscience, Copernicus Institute of Sustainable Development, Utrecht University, reports a relationship with Faculty of Geosciences, Copernicus Institute of Sustainable Development - Utrecht University that includes: employment. Gokhan Sahin has patent pending to There is no patent. there is no conflict of interest. If there are other authors, they declare that they have no known competing financial interests or personal relationships that could have appeared to influence the work reported in this paper.
